# Supplementary material for: Significantly Reduced Alanine Aminotransferase Level Increases All-Cause Mortality Rate in the Elderly after Ischemic Stroke
Source: Int J Environ Res Public Health. 2021 May 5;18(9):4915. doi: 10.3390/ijerph18094915 (PMC8125228; doi:10.3390/ijerph18094915)
Supplement: Supplementary file 1 [file ijerph-18-04915-s001.zip › ijerph-1167769-supplementary.pdf]

Table S1. Univariate analysis. Hazard ratio and 95% CI for all-cause mortality, according to the selected risk factors.

	Hazard Ratio	95% Confidence Interval		*p*-Value
		Lower	Upper	
Age (years)	**1.08**	1.04	1.20	<0.001*
Sex (Reference; female)				0.019
- Male	1.92	1.12	3..2	
Initial NIHSS at admission	1.10	1.06	1.14	<0.001*
Thrombolytic Treatment	0.68	0.31	1.51	0.343
SBP at admission	1.00	0.99	1.01	0.392
SBPV during first 24 h	1.01	1.00	1.03	0.125
TOAST				0.618
- Large-artery atherosclerosis (Reference)				
- Cardioembolism	0.83	0.46	1.49	0.522
- Small-vessel occlusion	0.94	0.45	1.96	0.877
- Stroke of other determined etiology	0.00	0.00	0.00	0.967
- Stroke of undetermined etiology	0.43	0.16	1.22	0.113
Location (Reference; both site)				0.176
- Infratentorial	2.44	0.73	8.12	0.147
- Supratentorial	2.79	0.94	8.26	0.064
Hemispheric localization (Reference; both side)				0.031*
- Left	0.41	0.21	0.81	0.010*
- Right	0.61	0.31	1.20	0.153
Laboratory findings at diagnosis				
- Extremely low ALT (<10 U/L)	2.73	1.50	4.98	<0.001*
- Low albumin (<3.5 g/dL)	1.56	0.84	2.92	0.161
- Low Hb (<11 g/dL)	1.17	0.632	2.15	0.622
- Random Glucose (Reference; 3.7~7.3 mmol/L)				0.044*
- Hyperglycemia (>7.3 mmol/L)	0.88	0.49	1.58	0.662
- Hypoglycemia (<3.7 mmol/L)	16.05	1.76	146.07	0.014*
- Creatinine (mg/dL)	1.59	1.06	2.38	0.024*
- ESR (mm/h)	1.01	0.994	1.02	0.412
- NLR	0.97	0.911	1.03	0.304
- Total cholesterol (mg/dL)	1.00	1.00	1.01	0.79
BMI (Reference; 18.5–24.9)				0.416
- Underweight (<18.5)	1.57	0.72	3.42	0.259
- Overweight (25.0–29.9)	0.71	0.38	1.32	0.281
- Obese (≥30)	1.09	0.38	3.17	0.868
Atrial fibrillation	0.89	0.50	1.60	0.703
DM	1.47	0.85	2.54	0.167
Hypertension	01.06	0.64	1.75	0.814
Coronary artery occlusive disease	1.14	0.60	2.16	0.698
Cancer history	0.88	0.33	2.35	0.793
Smoking history	1.08	0.53	2.19	0.828
Alcohol consumption	0.75	0.37	1.53	0.424

Values, mean ± standard deviation; ALT, Alanine aminotransferase; BMI, Body Mass Index; DM, Diabetes mellitus; ESR, Erythrocyte Sedimentation Rate; Hb, Hemoglobin; NIHSS, National Institute of Health Stroke Scale; NLR, Neutrophil-Lymphocyte Ratio; SBP, Systolic Blood Pressure; SBPV, Systolic Blood Pressure Variability; TOAST, Trial of ORG 10172 in Acute Stroke Treatment; * *p*

< 0.05.
